# Building a Gender-Bias-Resistant Super Corpus as a Deep Learning Baseline for Speech Emotion Recognition

**DOI:** 10.3390/s25071991

**Published:** 2025-03-22

**Authors:** Babak Abbaschian, Adel Elmaghraby

**Affiliations:** Computer Science and Engineering Department, University of Louisville, Louisville, KY 40292, USA; adel.elmaghraby@louisville.edu

**Keywords:** speech emotion recognition, deep learning, LSTM, CNN, gender bias, fairness, speech emotion database, transfer learning, transformers

## Abstract

The focus on Speech Emotion Recognition has dramatically increased in recent years, driven by the need for automatic speech-recognition-based systems and intelligent assistants to enhance user experience by incorporating emotional content. While deep learning techniques have significantly advanced SER systems, their robustness concerning speaker gender and out-of-distribution data has not been thoroughly examined. Furthermore, standards for SER remain rooted in landmark papers from the 2000s, even though modern deep learning architectures can achieve comparable or superior results to the state of the art of that era. In this research, we address these challenges by creating a new super corpus from existing databases, providing a larger pool of samples. We benchmark this dataset using various deep learning architectures, setting a new baseline for the task. Additionally, our experiments reveal that models trained on this super corpus demonstrate superior generalization and accuracy and exhibit lower gender bias compared to models trained on individual databases. We further show that traditional preprocessing techniques, such as denoising and normalization, are insufficient to address inherent biases in the data. However, our data augmentation approach effectively shifts these biases, improving model fairness across gender groups and emotions and, in some cases, fully debiasing the models.

## 1. Introduction

With the introduction of mainstream deep learning methods, the doors to solving more complex digital signal processing problems, such as Automatic Speech Recognition (ASR) and Speech Emotion Recognition (SER), have opened. Reviewing research published in the past decade shows a positive trajectory of efficiency and accuracy for each year [1].

One of the key drivers behind SER development is the effort by major tech companies to create robust solutions for this challenge. Notable examples include Amazon Alexa, Google Assistant, Apple Siri, and Microsoft Cortana [2]. There has been an influx of research on SER, from proposals to create better training sets to methods that increase accuracy and to others that make the systems more robust and reliable in real situations.

The rationale behind these endeavors stems from the anticipation that advancements in SER will significantly enhance the landscape of human–computer interaction. The pivotal role of Speech Emotion Recognition lies in its potential to facilitate the development of systems that are capable of comprehending human commands with greater acuity and responding adeptly across diverse scenarios. Noteworthy instances of application include the optimization of interactions between smart speakers, virtual assistants, and end users. In particular, SER proves instrumental in refining the quality of exchanges within speech-to-text applications, addressing the challenges of informal language structures that deviate from conventional grammar and syntax. This is exemplified when written sentences may not accurately convey content due to the absence of appropriate intonation, such as in the case of polar (Yes/No) questions, as illustrated by the query “You pet the cat?” [3]. SER also has benefits in psychotherapy, customer service, remote learning, self-driving vehicles, and more [1].

As previously mentioned, earlier research primarily focused on Automatic Speech Recognition (ASR) and traditional machine learning techniques. However, these approaches demonstrated limited performance, with reported accuracies typically in the range of 70% [3,4]. However, the newer publications focusing on deep learning are generating better results, reporting 95% [5], 93% [6], 95.5% [7], and 97% accuracy [8].

In addition to the methods discussed, many databases have been introduced for SER. General categories of emotional speech databases include natural, semi-natural, and simulated databases [1].

All things considered, the reliability of SER models faces challenges in terms of fairness, robustness, and accuracy when confronted with environmental noise, out-of-distribution test settings, and gender bias. Environmental noise, such as background sounds, can introduce variability and hinder the model’s performance. Out-of-distribution test settings, where the data differ significantly from the training set, threaten the generalization capabilities of SER models. Additionally, gender bias can manifest in disparities in the recognition accuracy across different gender groups. Recognizing these vulnerabilities, we are exploring normalization techniques, noise reduction strategies, and the utilization of a comprehensive super corpus to enhance the fairness, robustness, and overall accuracy of SER models in diverse and challenging scenarios.

### 1.1. Contribution

As mentioned above, despite the notable achievements of deep neural networks (DNNs), their efficacy is contingent upon the characteristics of the training data, and concerns persist regarding their generalization capabilities. While recent attention has been directed toward assessing the fairness and robustness of DNNs in computer vision and natural language processing, to the best of our knowledge, we are the first to explore the robustness of SER models to the speaker’s gender and out-of-distribution test samples. Specifically, our contribution includes the following:

1. Building and evaluating a series of modern deep-learning-based architectures, establishing a new baseline that outperforms older benchmarks across accuracy, F1 score, precision, and recall metrics while maintaining balanced performance across datasets and preprocessing strategies.
2. Introducing a super corpus that augments the sample pool and diversifies the datasets, enabling broader applicability and robustness.
3. Conducting a detailed examination of model robustness concerning speaker gender and cross-corpora scenarios.
4. Finally, we will provide a comprehensive discussion on how our proposed super corpus, coupled with various preprocessing strategies, contributes to improving generalization, mitigating gender bias, and enhancing the robustness of the SER models to the out-of-distribution data samples.

The resulting models and datasets serve as a foundational baseline for future research in this domain.

### 1.2. Organization

The rest of the paper is organized as follows: Section 2 revisits related works in cross-corpus SER and fairness discussions in speech processing. Section 3 sets up the problem and formalizes the approach for crafting super corpora. Section 4 discusses the datasets and the examination metrics and models. We will present and discuss the results in Section 5; the last section concludes the paper.

## 2. Related Works

The known problems of generalization, and limited data available to train SER models has driven many to try creative methods such as augmenting data or cross-corpus training. In this chapter, we will review some of the works on mitigating data limitations and the demographic bias of SER systems. 

Additionally, one common approach to mitigating the lack of inductive biases, such as architectural choices or strong priors, in deep learning models is to expand the amount of training data available [9,10]. Welling [9] argues that increasing data and computational resources can, to some extent, compensate for the absence of explicit model structures or biases. Similarly, Baxter [10] discusses how inductive bias plays a critical role in enabling generalization, particularly when data is limited. However, as Goyal and Bengio [11] point out, while various forms of inductive biases can serve as substitutes for more, or less data, their impact may diminish in scenarios where large-scale datasets are available. This suggests that the evaluation and implementation of inductive biases become especially valuable in transfer learning settings, where models must generalize to new distributions with limited available examples.

In an early effort at data augmentation, and cross-corpus challenges Zhang et al. [12] r explored the effectiveness of unsupervised learning for data augmentation. Their study utilized six emotional speech corpora of ABC [13], AVIC [14], DES [15], Enterface [16], SAL [17], and VAM [18]. Since SAL and VAM are annotated using arousal/valence dimensions, while the remaining datasets are labeled with categorical emotions, they mapped the categorical labels to corresponding arousal and valence values to unify the emotional representations across datasets.

For feature extraction, they employed the openEAR toolkit [19], selecting 39 out of the available 56 functional acoustic Low-Level Descriptors (LLDs). Additionally, they applied mean normalization to each dataset to ensure zero mean across features, aiming to reduce corpus-specific biases.

Their evaluation followed a cross-corpus leave-one-out strategy, conducting three main experiments:

1. Multi-corpus supervised training—They combined three datasets for training and tested on a separate corpus.
2. Semi-supervised learning—A supervised model was trained on three labeled datasets, then further adapted using two additional unlabeled datasets, and finally tested on a remaining dataset.
3. Comprehensive cross-corpus training—They trained on five datasets and tested on the remaining one.

Their findings demonstrated that incorporating unlabeled data into the multi-corpus training process improved recognition accuracy. However, they observed that the performance gain from adding unlabeled data was approximately half of the improvement achieved when adding an equivalent amount of labeled data.They employed the openEAR toolkit [19] to extract features and retain 39 of the (LLDs). As an extra step in their preprocessing, they normalized all databases to zero means. Finally, to evaluate their experiment, they followed a cross-corpus leave-one-out strategy.

In 2019, Milner et al. [19] investigated cross-corpora SER by incorporating a bidirectional LSTM with an attention mechanism. Their research investigates information transfer from acted databases to natural databases. Moreover, they have also looked into domain adversarial training (DAT) and out-of-domain (OOD) models and considered adapting them.

Their network is a triple attention network consisting of a BLSTM, the attention architecture, and the emotion classifier at the end. For domain adversarial training, they also considered adding a domain identifier to the training set that teaches the model how it is managing with each dataset. In their work, they used two acted datasets, eNTERFACE and RAVDESS, one elicited dataset, IEMOCAP, and one natural dataset, MOSEI.

This study concludes that when testing cross-corpus, the matched results outperform the mismatched results, and the model trained on simulated datasets generally achieves the best mismatched performance. They also discussed that the model trained on multi-domain performs better than all of the other mismatched models due to more generalization resulting from having a larger dataset. They showed in their results that adding the domain information does not help the multi-domain model to generalize better, but training with more other domains helps to improve the mismatched results.

In 2021, Wisha et al. [20], worked on a cross-corpus, cross-language ensemble method to detect emotions from four languages. They used Savee (English) [21], URDU (Urdu) [22], EMO-DB (German) [23], and EMOVO (Italian) [24] in their research. However, their study only investigates binary valence. To train their classifier, they used spectral features such as 20 Mel-Frequency Cepstral Coefficients (MFCCs) and prosodic features defined in eGeMAPS [25]. The classifiers that create the ensemble are SVM with a Pearson VII function-based Universal Kernel, a random forest with ten trees, and a C4.5 algorithm decision tree.

In their in-corpus evaluations, they reported accuracy improvements of 13% on the URDU dataset, 8% on EMO-DB, 11% on EMOVO, and 5% on SAVEE. In cross-corpus experiments, they achieved the following accuracy gains: 2% when training on Urdu and testing on German, 15% when testing on Italian, 7% when testing on Urdu while training on German, 3% when training on Italian, and 5% when training on English.

In 2021, Braunschweiler et al. [26] investigated the impact of cross-corpus data augmentation on model accuracy. In their research, they incorporated a network with six layers of CNN, a Bidirectional LSTM model with two layers of 512 nodes, and four fully connected layers followed by an attention mechanism. The databases used in this research are IEMOCAP [27], RAVDESS [28], CMU-MOSEI [29], and three in-house single speaker corpora, named TF1, TF2, and TM1; F and M stand for female and male, respectively. The classes they chose to recognize using their model were angry, happy, sad, and neutral. To increase variability in their samples and improve their model generalization, they also applied variable speed, volume, and multiple frequency distortions such as bass, treble, overdrive, and tempo changes to the samples.

Their investigation shows that in situations where the model was trained with one database and tested with another, they have an accuracy decline of 10–40%. They further discuss that their results were improved when the model was trained with more than one corpus and tested on one of the corpora in the training set, except for their single-speaker datasets. The last result that they report is a 4% gain in accuracy with additional data augmentation.

Later, in 2022, Latif et al. [30] introduced an adversarial dual discriminator (ADDi) network trained on cross-language and cross-corpora domains. They claim that their model improves the performance of the state-of-the-art models. Their model contains an encoder, a generator, and a dual discriminator. In their model, they map the data to a domain-invariant latent representation. The generator uses the result of the encoder to generate target or source domain samples and the two adversarial discriminators that, in combination with the generator, tune the domain invariant representation to minimize the loss function. The generator and the encoder act as decoders to construct the input samples.

In their self-supervised training process, they introduce synthetic data generation as a pretext task that helps to improve domain generalization. As a byproduct, synthetic emotional data are produced that can augment the SER training set and help with more generalization.

They further discuss that introducing the ADDi network improves cross-corpus and cross-language SER without using target data labels. They also add that their model significantly improves these by feeding partial target labels. They also claim that with the help of the self-supervised pretext task, they can achieve the same performance by training their ADDi network with 15–20% less training data.

Regardless of the accuracy of deep learning models, their robustness to changes in data distribution and making fair decisions is still open research. In 2020, Meyer et al. [31] published Artie Bias Corpus as the first English dataset for speech recognition applications with demographic tags, age, gender, and accent curated from Mozilla Common Voice corpus [32]. Additionally, they published open-source software for their dataset to detect demographic biases in ASR systems.

Similarly, in 2021, Feng et al. [33] quantified the bias in the state-of-the-art Dutch Automatic speech recognition (ASR) system against gender and age. Their work reported the bias regarding word error rates (WER). They concluded that the ASR system studied had a higher WER for male Dutch speakers than for female speakers.

Lastly, in 2022, a team of researchers from Meta [34] released a manually transcripted dataset containing 846 h of corpus for fairness assessment of ASR and facial recognition systems across different ages, genders, and skin tones. According to their results, several ASR systems lack fairness across gender and skin tone and have higher word error rates for specific demographics.

In 2024, several notable works emerged related to the predictive performance of SER, further expanding the landscape of SER research. Hasan et al. [35] introduced EmoFormer, a hybrid Transformer-CNN model for text-independent Speech Emotion Recognition. This research demonstrates the growing interest in combining transformer-based architectures with CNNs for improved performance. Similarly, Avro et al. [36] proposed EmoTech, a multi-modal SER framework that leverages multi-source low-level acoustic information with a hybrid recurrent network. Islam et al. [37] also explored deep convolutional neural networks (CNNs) to enhance emotion recognition accuracy, reflecting the continuous advancements in deep learning methods for SER. These recent studies highlight promising architectural innovations that align well with our future research direction in mitigating data and bias challenges for SER systems.

## 3. Problem

The current challenge in the field pertains to the resilience of deep learning models against out-of-distribution data instances and demographic biases. This matter persists as an unresolved concern. Our approach involves the utilization of pre-existing open-source datasets to enhance the generalizability of established SER methodologies. Furthermore, we endeavor to alleviate biases directed towards particular speaker genders whenever feasible. We assert that the augmentation of datasets within the realm of deep learning models for SER holds substantial potential, mainly when such augmentation is cost-effective and maintains the explicability of the end-to-end process. Within our dataset repository, we permit the augmentation of each dataset with all others. Notably, we introduce a specific case wherein solely simulated datasets undergo augmentation. This is motivated by their shared attributes, such as a controlled noisy environment and a predefined set of speakers. Additionally, our experimental framework examines the impacts of noise reduction and normalization.

## 4. Experimental Setup

In this section, we introduce our approach to generating super corpora. Additionally, we explain how we built our baseline setup based on a wide range of available architectures for deep-learning-based SER.

Before detailing the process, our experimental setup hardware and software is given as follows.

- Software:
  ○ Python Programming Language, version 3.11;
  ○ Pandas Library, version 2.1.4;
  ○ Keras Framework, version 3.0.5;
  ○ Pytorch, version 2.1.2;
  ○ Tensorflow, version 2.13.1.
- Hardware:
  ○ Intel Core i7 workstation with 64 GB of RAM (General Data processing);
  ○ AWS EC2 p3.16xlarge, with 64 core vCPUs, 488 GB RAM, and 8 NVIDIA Tesla V100 16 GB GPUs (Deep learning model training).

### 4.1. Super Corpora

As previously stated, the performance of SER systems experiences degradation when confronted with out-of-distribution samples. Furthermore, as demonstrated in Section 5, the performance of these systems varies inconsistently across different speakers’ genders. In our proposed solution, we endeavor to address these issues by mitigating the impact of data distribution disparities. This is achieved through the augmentation and amalgamation of diverse datasets, yielding a comprehensive dataset called a “super corpus”.

Datasets in SER are classified into three categories: Natural, Semi-Natural, and Simulated. Natural datasets are derived from authentic speech instances from diverse contexts such as news, online talk shows, and customer service call recordings. Labeling such datasets is inherently challenging due to the ambiguity of speaker intentions and the potential for varied listener interpretations of emotions. Consequently, the labeling process necessitates a sizable cohort of annotators and a structured voting system to determine emotional labels. Another inherent challenge with natural datasets lies in the dynamic nature of emotions within spontaneous speech. For instance, in a customer service call, emotions can transition rapidly from a neutral state to frustration or anger within seconds. This fluidity poses difficulties in precisely labeling utterances or even entire sentences, constituting a complex and subjective task [1]. Examples of natural datasets include Vera Am Mittag (VAM) [18], and FAU Aibo [38].

Semi-natural datasets are created based on predefined scenarios and plots, and then one or more voice actors execute them. The emotional expressions within this dataset category are not strictly organic and may sometimes be exaggerated. Nonetheless, the advantage lies in achieving heightened control over the dataset, as the intended emotions are known, rendering the labeling process more dependable. However, challenges persist within this dataset paradigm, particularly concerning the dynamic nature of emotions and the intricate task of labeling utterances [1]. IEMOCAP [27], Belfast [39], and NIMITEK [40] are examples of this type of dataset.

The third dataset category, Simulated, is constructed from a set of emotionally neutral sentences enunciated by voice actors who infuse various emotions into their delivery. The employment of emotionally neutral sentences imbued with diverse emotional expressions serves dual purposes. Firstly, it prevents the acquisition of emotionally biased sentences being learned by machine learning models, thereby mitigating the risk of triggering responses based solely on, for instance, the identification of emotion-related keywords such as “angry” within a speech signal. Secondly, the repetition of identical sentences articulated with different emotions ensures that the classifier model remains impervious to the semantic content of the sentences, thereby facilitating the isolation of the shared direct current (dc) component in the convoluted signal field [1]. EMO-DB (German) [23], DES (Danish) [15], RAVDESS [28], TESS [41], and CREMA-D [42] are examples of this type of dataset.

To systematically select integrated datasets, we established our criteria set as follows: the primary criterion for dataset selection is language. Given the variation in how emotions are expressed through speech across different languages, we exclusively considered datasets in the English language. Subsequently, for result comparison, we opted for datasets associated with multiple models substantiated by published papers. Furthermore, given our focus on addressing the challenge of exposure to out-of-distribution data samples, we prioritized datasets characterized by a substantial volume of samples and a diverse spectrum of emotional expressions, encompassing not only positive or negative sentiments. Plus, we planned to utilize simulated-only and semi-natural datasets to address labeling challenges, variations in utterance size, and linguistic nuances related to emotional content. Therefore, from the list of open-sourced datasets in English, we chose three simulated English datasets, namely RAVDESS, TESS, and CREMA-D, plus the widely used semi-natural dataset IEMOCAP. Ultimately, we abstained from employing augmentation processes rooted in deep learning to maintain interpretability within our methodology. Instead, we exclusively leveraged the extant data samples to augment each dataset. This will facilitate subsequent extensions of our robustness evaluations to encompass more intricate scenarios, notably including assessments of robustness against adversarial attacks, which we will address in future works.

From the selected databases, which feature a variety of emotions, we found that four emotions, Happiness, Anger, Sadness, and Neutral, are present in all of them. We decided to use these samples for our project. In Table 1, we present a summary of each dataset. Since we aim to investigate the performance of models with respect to bias against the speaker’s gender, the presented statistics are divided based on two groups of speakers, male and female.

### 4.2. Building the Super Corpora

To construct our experimental corpus, we utilized a selection of databases, each comprising PCM (Pulse Code Modulation)-encoded WAV files. However, the encoding formats, sample rates, and other audio characteristics varied significantly across the databases. For instance, the CREMA-D database had a sample rate of 16 kHz, while others used 48 kHz. Additionally, two databases were recorded in stereo, while the remaining two were mono. We resampled the audio files to 16-bit, 16 kHz, and mono signals to ensure uniformity across all datasets.

Upon reviewing file sizes and utterance durations, we observed that several utterances in the IEMOCAP database were shorter than one second. These short samples presented challenges even for human listeners in reliably identifying emotions. Consequently, we excluded all samples with less than one-second durations from our dataset.

Further analysis of the files revealed significant statistical differences across the datasets, including variations in average mean, DC component (Mean Signal Offset), peak amplitude, amplitude range, Signal-to-Noise Ratio (SNR), voicing characteristics such as Zero-Crossing Rate (ZCR), and prosodic features. Notably, voicing and prosodic features are closely linked to the emotional content of speech signals.

In contrast, features like the DC component, Root-Mean-Square (RMS) energy, and SNR primarily create energy-related signal denormalization effects. For example, normalizing the audio could reduce the energy in high-frequency samples (e.g., Angry or Happy emotions) or mitigate noise interference in low-energy samples (e.g., Sad emotions). Moreover, the same normalization will reduce the relative prominence of high-energy components (e.g., transients or spikes) across all frequencies. As a result, their perceptual prominence will change. Similarly, spectral noise reduction will attenuate energy at the signal’s lower and higher frequency margins as an unwanted effect.

One of the key features in gender identification is the signal’s high- and low-frequency energy content [43]. Consequently, these preprocessing methods are likely to reduce gender-specific characteristics in speech. Based on this observation, we hypothesized that normalization and spectral noise reduction would remove more gender-specific discriminative content than emotional information from the speech signals.

We applied preprocessing methods, including RMS normalization and spectral noise reduction, to test this hypothesis and evaluate their effects on classification performance and bias. We implemented four preprocessing schemes and applied them to all datasets:

1. No preprocessing (Raw);
2. RMS Normalized;
3. Noise Reduced;
4. RMS Normalized and Noise Reduced.

In the next step, we created a series of MFCC representations for all datasets while retaining the original pulse-code modulation (PCM) in WAV file format. For MFCC generation, we experimented with different window sizes in both time and frequency domains. Timewise, we used shorter window sizes, such as 25 ms, and longer durations approaching one second. Frequency-wise, we generated representations with 13 coefficients (including delta and delta-delta) and 64 coefficients (also with delta and delta-delta).

Additionally, we experimented with various database combinations. These included the following:

1. Merging all samples from all databases into a single bucket (All);
2. Using only simulated database samples (Simulated Only);
3. Keeping each database separate.

**Figure 1:**
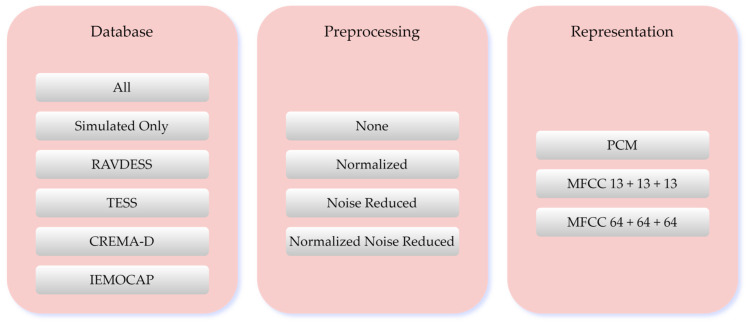
Each combination of databases had gone through all of the preprocessing options, creating four variations. Each of the variations is then represented by the representation schemas.

This setup resulted in six database combinations. By applying all preprocessing schemes across these combinations, we built a total of 72 variations, combining the six primary databases for analysis. Figure 1 illustrates all the database combinations used in this investigation.

### 4.3. Deep-Learning-Based SER

Creating a versatile baseline is essential to covering a wide range of diverse approaches to SER. Therefore, we have chosen several deep learning methods, specifically ANN-based, CNN-based, and LSTM-based, to cover various methods that are applicable to MFCC preprocessed speech datasets. In Table 2, we present a summary of the networks we have examined, and we will explain the results in Section 5.

The architecture names in the first column of Table 2 follow a systematic notation to represent the layers used in each model. For instance, “3XCNN1D 1XLSTM 2XDENSE” refers to a model that begins with three one-dimensional convolutional neural network (CNN) layers, followed by a single Long-Short-Term Memory (LSTM) layer, and concludes with two fully connected dense layers. As clarified in the literature [1], each layer type serves a specific purpose in processing the speech data, which we briefly revisit in the following sections.

#### 4.3.1. One-Dimensional Convolutional Layers (CNN1D)

In SER, CNN1D captures spatial patterns across features, making them ideal for analyzing temporal sequences like MFCCs, which encapsulate the frequency-time patterns in speech signals.

#### 4.3.2. One-Dimensional Temporal Convolutional Networks (TDCNN1D)

Architecturally, TDCNN1D is similar to 1D CNNs but tailored to capture longer dependencies using dilated convolutions. These enable the network to look further back in the sequence without substantially increasing the computational cost.

#### 4.3.3. Long Short-Term Memory (LSTM)

LSTM layers are recurrent neural networks (RNNs) designed to capture long-term dependencies and temporal relationships, which are critical for speech-related tasks, where context over time can influence emotion detection.

#### 4.3.4. Dense

Finally, dense (fully connected) layers integrate the features learned by previous layers, enabling the model to make final classifications or predictions.

Each model configuration in Table 2 varies based on the dataset used (e.g., IEMOCAP, CREMAD) and preprocessing techniques applied to the MFCCs, such as denoising or normalization. These variations help us understand the model’s robustness and adaptability across different datasets and preprocessing choices, providing a comprehensive foundation for SER research.

### 4.4. Downstream Bias

Female speakers have been found to convey their emotions more expressively than male speakers, which could result in inconsistent performance if the classifier is used in real-world applications. The empirical true positive rate (*TPR*) estimates the probability that the classifier accurately identifies a person’s emotion from their speech. Following previous research [44,45], we measured downstream bias by examining the empirical TPR gap between speeches for each gender set. First, the following must be defined:

$$TPR_{y,g}=P\left[\hat{Y}=y|G=g,Y=y\right]$$

where *g* is a set of genders, and *y* is an emotion. Y and $\hat{Y}$ are the true and predicted emotions, respectively. Then, TPR bias (TPB) is defined as follows:

$$TPB_{y}=\frac{TPR_{y,Female}}{TPR_{y,Male}}$$

where if a classifier predicts “Angry” for a male speaker much more often than for a female speaker, the *TPR* ratio for the “Angry” class is low.

## 5. Results and Discussion

This section presents the results of employing our augmentation process to craft super corpora on the deep-learning-based SER models. We show both predictive performance-related experiments on all datasets and gender-specific generalization experiments. When developing SER systems, the final model is trained and tested on a specific dataset. In this situation, the generalization and predictive performance of the developed model are constrained to the dataset. However, based on our experiments in this section, we demonstrate that the models’ performances are inconsistent in inner-corpora and cross-corpora settings. Additionally, even the best models, i.e., those with high accuracy and F1 scores, are biased, and their performance is inconsistent across different speaker genders. Ultimately, our proposed augmentation approach effectively improves the cross-corpora performance, also known as exposure to out-of-distribution generalization, and mitigates gender bias.

We described the construction of our Super Corpora experiment in Section 4.2, “Building the Super Corpora”. This process resulted in 72 combinations derived from the six primary databases, along with more than 13 models used to conduct the experiments. After some experimentation and comparing the results, as one of our objectives was adding a limited computation overhead, we continued working with the MFCC window of (120, 39), with about 600 ms audio, and 13 + 13 + 13 coefficients with a 25% overlapping window. We discontinued the use of the 64 + 64 + 64 MFCCs, as their performance gain was limited compared to complexity overhead. Also, we explored spectrogram utilization in our initial experiments, as Wani et al. suggested [46]. However, spectrogram-based experiments are not computationally efficient and suffer from implicit biases.

We conducted extensive experimental evaluations across a range of network architectures and varying model sizes applied to each dataset. However, in this section, we only report the performance of top networks on each dataset and finally extend our augmentation experiment on them. It is noteworthy that DSCNN2D, as SER state-of-the-art architecture, has not consistently outperformed other architectures in our experiment.

Additionally, the gender-bias hypothesis in the form of downstream bias is measured for each dataset both separately and combined. This allows us to investigate whether the bias is influenced by the dataset specification or implicitly by the network architecture. The bias hypothesis here is measured using the *TPB* metric (described in Section 4.4) as well as the differences in predictive metrics (accuracy, F1 score, precision, recall, and confusion matrix) between different groups of speakers and emotions. Moreover, we investigate how models trained with a particular dataset using different preprocessing approaches, normalization, and noise reduction are vulnerable when exposed to out-of-distribution data samples. Finally, we present how our proposed super-corpus improves each deficiency and the emotional confusion of these models for female and male speakers.

Table 2 presents the overall accuracy and F1 score, precision, and recall of each top architecture across individual datasets and the proposed super corpus in four preprocessing scenarios. As mentioned in the Section 4.2, “Building the Super Corpora”, in our experiments, we refer to the super corpora that have been augmented by using all datasets as “All”, and, as the name implies, “Simulated only” refers to the super corpora that have incorporated RAVDESS, TESS, and CREMA-D in the augmentation process.

### 5.1. Deep Learning Architectures and Their Performance

As mentioned in the previous section, IEMOCAP is a semi-simulated and relatively large dataset. As can be found from the results, SER models can benefit from our data augmentation approach to improve their generalization and demonstrate better predictive performance at no computation cost since the batch size and training epochs have been fixed for all of the reported results. Notably, having the best performance over the TESS dataset can be explained by its unique design, smaller spoken content variation, and only two female speakers uttering all the samples. Apart from the TESS dataset, the “simulated only” augmented dataset effectively outperforms all others, regardless of the preprocessing schemes.

At the beginning of this study, one objective was to establish a modern baseline for the SER models, ensuring that future comparisons are not limited to state-of-the-art models from two decades ago. To this end, Table 3 summarizes the previously published state-of-the-art results across the model architecture families that we implemented and compares our models and their corresponding best-performing counterparts. Our models demonstrate superior predictive performance compared to results reported in prior research. Moreover, the models presented in this work are assessed for generalization and fairness. In contrast, many earlier works, including those referenced in Table 3, primarily report accuracy alone, offering a limited perspective on system performance.

**Figure 2:**
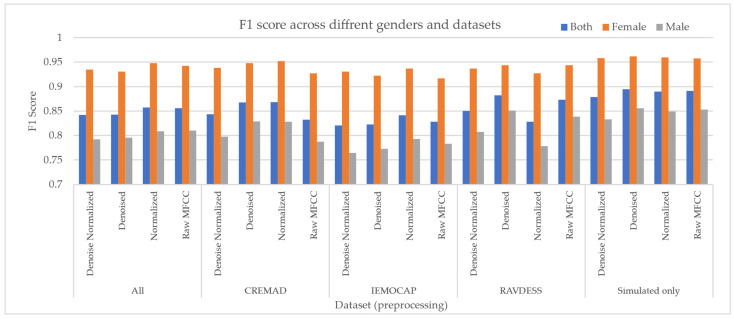
Cross-gender performance of models on each dataset. For each dataset, the effect of normalization and denoising has been reported.

In the next step, we separated the data samples from female and male speakers to assess gender-related bias and compare model performance for each group. As shown in Figure 2, the models exhibited inconsistent and non-robust performance with respect to the speaker’s gender. However, under a constrained training setup—using fixed training epochs and batch size—and by applying our augmentation approach, we observed improvements not only in predictive performance but also in model robustness across genders. This led to a reduction in bias, as measured by the difference in F1 scores Δ(F_1 score), among the “Both”, “Male”, and “Female” speaker groups. Additionally, our Super Corpora augmentation method proved effective regardless of traditional preprocessing techniques, helping to lower the overall computational cost of SER systems. 

**Figure 3:**
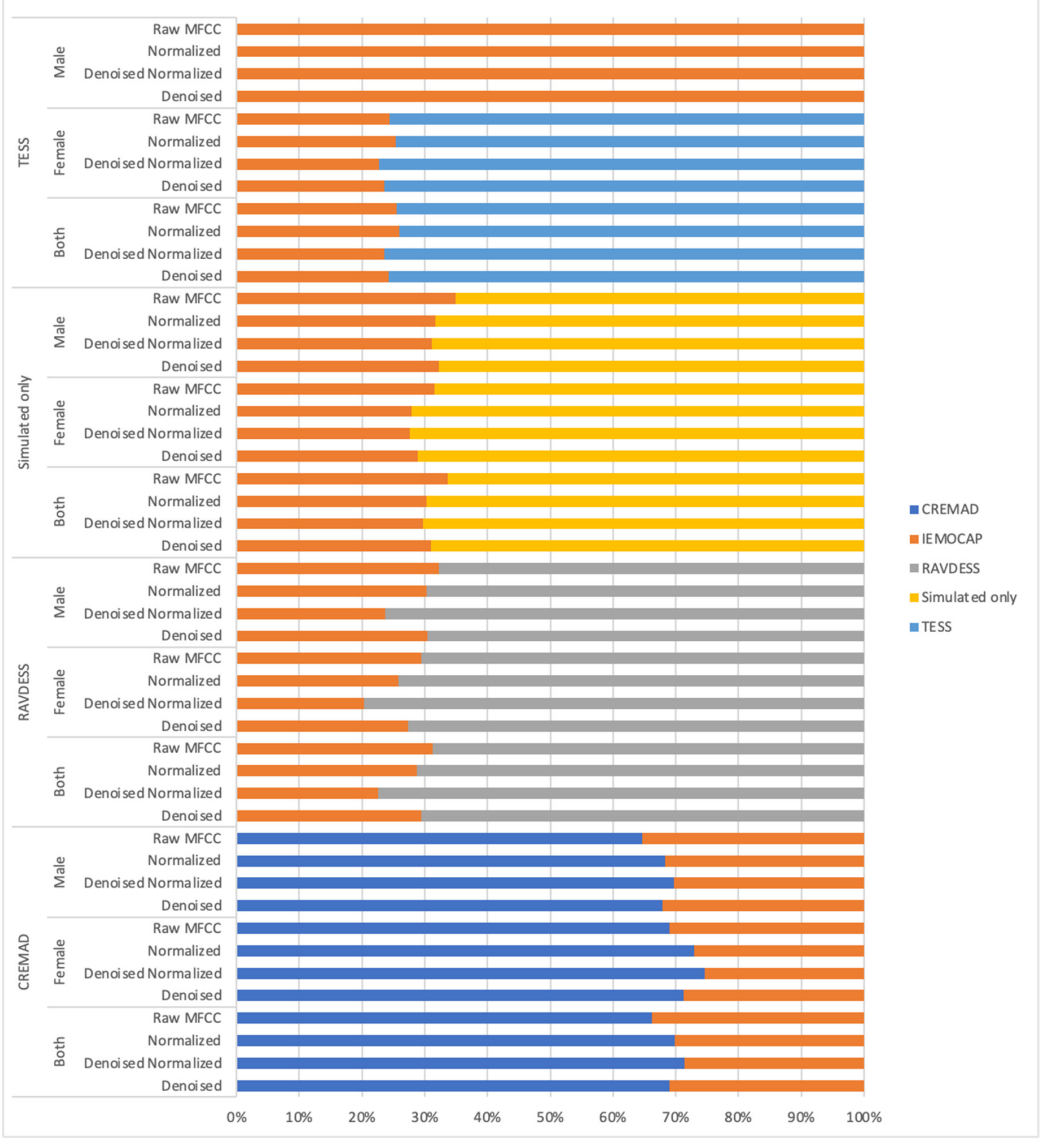
Weighted F1 scores of the top model trained on different dataset variations, evaluated on in-domain (same dataset) and out-of-distribution (IEMOCAP). The *Y*-axis represents different training dataset variations (original, denoised, normalized, denoised and normalized, male-only, female-only, or both). At the same time, the *X*-axis shows the weighted F1 score (0–100). Each dataset variation includes two F1 scores: one for in-domain evaluation and one for IEMOCAP.

Figure 3 illustrates the cross-corpus effectiveness of our approach. We present the performance of the previously mentioned models when trained on individual simulated datasets (TESS, RAVDESS, IEMOCAP) and when trained on our “simulated only” Super Corpora. In each case, the models were tested on IEMOCAP, which was excluded from the training data.

As shown in Figure 3, all models—regardless of their inductive biases or alignment with specific training datasets—struggle when exposed to out-of-distribution data. This highlights a critical challenge when deploying models trained on a single dataset to real-world applications. However, models trained on a mixture of datasets, such as our Super Corpora, demonstrate improved generalization to out-of-distribution scenarios, even when they have not seen any data from the target distribution during training. 

### 5.2. Emotion-Level Bias Mitigation

**Figure 4:**
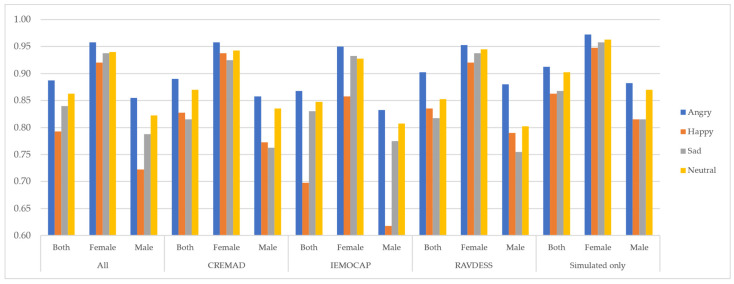
Average F1 score per database and gender across different emotions.

**Figure 5:**
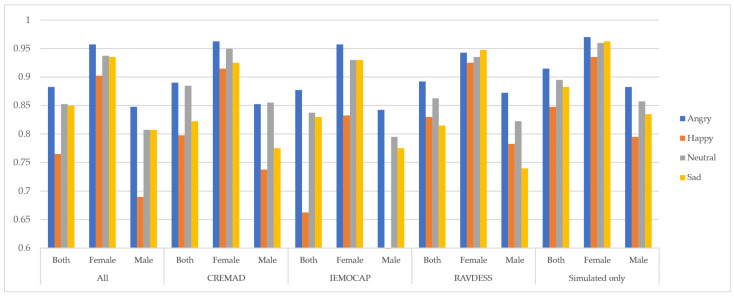
Average recall per database and gender across different emotions.

**Figure 6:**
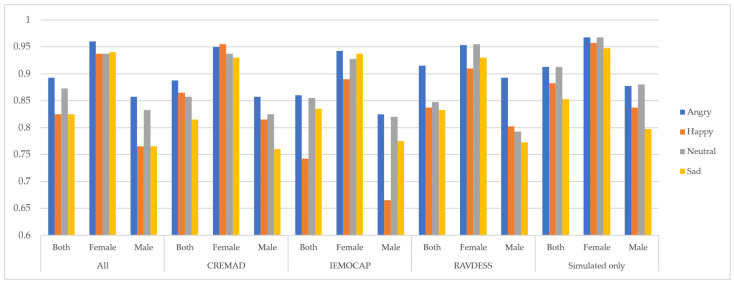
Average precision per database and gender across different emotions.

As shown in Figure 4, Figure 5 and Figure 6, our proposed data augmentation approach to creating a super corpus effectively mitigates biases at the emotion level. By integrating this augmented dataset, the overall fidelity of the data has improved significantly, specifically the inter-class performance variation, denoted by the following:

$$\Delta \left(M_{\left(i,j\right)}\right);\forall i,j\in \{emotions\}\;\mathrm{and}\;M\in \{F_{1},Recall,Precision\}$$

where emotions={Sad,Happy,Angry,Neutral} is effectively minimized.

This indicates that our approach reduces disparities across emotion categories, resulting in a more balanced and robust model performance. The improvement is evident through consistent evaluation metrics, reflecting enhanced model fairness and reliability.

### 5.3. Downstream Bias Measurment Results

Finally, in this section, we investigate whether downstream biases of the best models, as reported in Section 5.1, vary when trained with individual datasets compared to our augmentation super corpus. To measure downstream biases, we report $TPB_{y}$ for the best model (according to Table 1) across emotions of our datasets where, as mentioned in Section 4.4, if a classifier predicts, e.g., “Angry” for a male speaker much more often than for a female speaker, the TPR ratio for the “Angry” class is low. TPB = 1 implies an unbiased situation.

**Figure 7:**
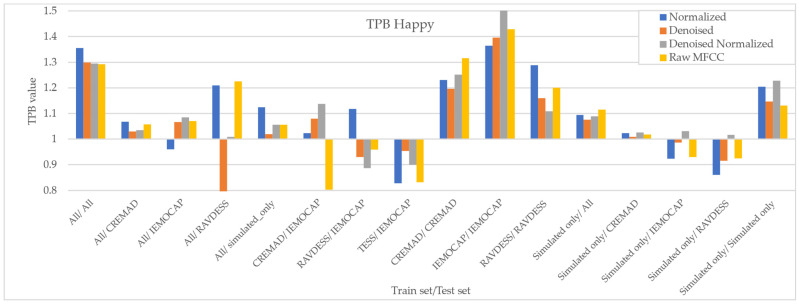
Measured downstream bias per ‘Happy’ emotions with respect to different preprocessing approaches and across different trainset/test sets.

**Figure 8:**
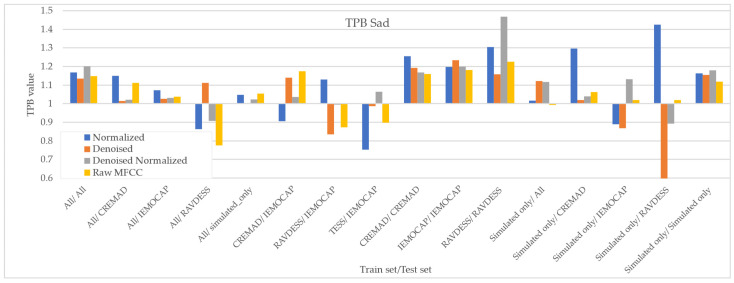
Measured downstream bias per ‘Sad’ emotions with respect to different preprocessing approaches and across different train/test sets.

**Figure 9:**
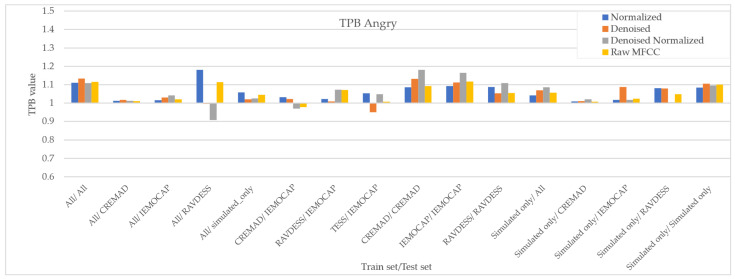
Measured downstream bias per ‘Angry’ emotions with respect to different preprocessing approaches and across different trainset/test sets.

**Figure 10:**
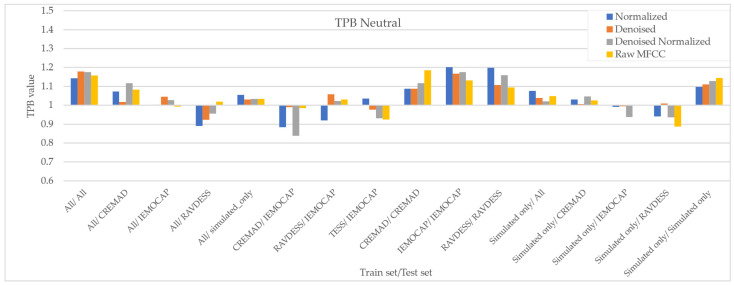
Measured downstream bias per ‘Neutral’ emotions with respect to different preprocessing approaches and across different trainsets/test sets.

As Figure 7, Figure 8, Figure 9 and Figure 10 illustrate, regardless of preprocessing methods and training datasets, we have witnessed lower TPB (0.85 < TPB < 1.2) for Angry and Neutral emotions compared to Happy and Sad (0.6 < TPB < 1.5). This means our best-performing classifier is transferring bias on Happy and Sad emotions, and the training data have a high impact on the performance of models in Happy and Sad detection. Additionally, as can be found from the reported Figures, different preprocessing approaches, i.e., Denoising, Normalization, or both, cannot shift the bias in models. However, our augmentation approach (Simulated only or All) can effectively shift the bias in the model and push biases in the desired direction and, in some cases, fully de-bias the models, e.g., Simulated only/All.

### 5.4. Limitations

While this study establishes a strong baseline for future research and highlights the significance of fairness, robustness, and reliability in Speech Emotion Recognition (SER) system evaluation, it is not without limitations. The primary limitations of this work can be categorized as follows:

#### 5.4.1. Methodological Constraints

As with any research, methodological constraints exist, including limitations in data collection, sample size, and measurement and labeling methods. Although we augmented our dataset by combining four publicly available datasets to enhance sample diversity, the overall dataset size remains relatively limited. This constraint affects the generalizability of our findings. Furthermore, at the time of this research, the availability of English-language SER datasets in the literature was scarce, which restricted our choices and may have influenced our results.

#### 5.4.2. Researcher Bias

A potential source of limitation in any study is researcher bias. However, in this work, we aimed to minimize its impact by hypothesizing that normalization and spectral noise reduction would reduce bias in our results. Contrary to our expectations, our findings did not support this hypothesis, suggesting that other factors may contribute to bias in SER models.

#### 5.4.3. External Constraints

Several external factors imposed limitations on this study. Resource constraints, including time and budget, restricted our ability to expand the study to additional languages and larger datasets. While several non-English SER datasets exist, incorporating them into our research would have required additional preprocessing and language-specific considerations, which were beyond the scope of this work. Additionally, more complex classification architectures could potentially enhance performance; however, given the limited number of training samples, we opted for a more constrained model to avoid overfitting and ensure reliable evaluation.

## 6. Conclusions

This paper introduces a low-cost data augmentation approach for SER systems, addressing critical gaps in their evaluation and performance. Through extensive experiments, we demonstrate that relying solely on F1 score and accuracy metrics provides an incomplete picture of SER systems’ effectiveness. Our findings emphasize the need for a holistic evaluation framework incorporating additional performance metrics and assessing fairness, robustness, and generalization. This approach highlights limitations in prior work, which often overstate system effectiveness by focusing narrowly on accuracy.

Our analysis reveals that even top-performing models exhibit inconsistent performance across gender groups (female, male), struggle with out-of-distribution samples, and show variability when analyzing different emotions within the same dataset distribution. Despite their strong F1 and accuracy metrics, these models continue to reflect biases inherent in the data. Moreover, whether used individually or in combination, standard preprocessing methods fail to address these biases adequately, such as denoising and normalization.

To tackle these challenges, we introduced a super corpus that significantly augments and diversifies the dataset pool, enabling broader applicability and enhancing robustness in SER models. Additionally, our detailed examination of model robustness concerning speaker gender and cross-corpora scenarios offers valuable insights into mitigating biases and improving generalization.

Our results further illustrate that the proposed augmentation approach, whether using simulated datasets alone or coupled with preprocessing strategies, effectively reduces or even eliminates bias in some cases, steering the model’s behavior in the desired direction. By demonstrating how our super corpus, when integrated with preprocessing strategies, enhances generalization, mitigates gender bias, and improves robustness to out-of-distribution data samples, we establish a comprehensive foundation for advancing SER systems.

This work establishes a strong baseline for future research. It underscores the importance of fairness, robustness, and reliability as essential dimensions of SER system evaluation alongside traditional performance metrics.

## Figures and Tables

**Table 1:** Number of samples per emotion (Happy, Neutral, Sad, Angry) generated by female and male speakers for each dataset. The total number of samples per dataset is the sum of the values in each row (e.g., RAVDESS has a total of 672 samples).

Dataset	Settings	Female Speakers					Male Speakers				
		Happy	Neutral	Sad	Angry	Total	Happy	Neutral	Sad	Angry	Total
RAVDESS	Simulated	96	48	96	96	336	96	48	96	96	336
TESS	Simulated	400	400	400	400	1600	0	0	0	0	0
CREMA-D	Simulated	600	512	600	600	2312	671	575	671	671	2588
IEMOCAP	Semi-Sim.	291	911	509	505	2216	304	797	575	598	2274

**Table 2:** Top architecture’s generalization and predictive performances across each individual and augmented dataset. Four preprocessing approaches have been employed per dataset.

Architecture	Dataset	Preprocessing	Accuracy	F1 Score	Precision	Recall
3XCNN1D 1XLSTM 2XDENSE	All	Raw MFCC	85.1%	0.856	0.861	0.851
3XCNN1D 1XLSTM 2XDENSE	All	Normalized	85.5%	0.857	0.861	0.855
3XCNN1D 1XLSTM 2XDENSE	All	Denoised	83.5%	0.843	0.853	0.835
3XCNN1D 1XLSTM 2XDENSE	All	Denoise Normalized	83.7%	0.842	0.847	0.837
3XCNN1D 1XLSTM 2DENSE	Simulated only	Raw MFCC	88.8%	0.891	0.895	0.888
3XCNN1D 1XLSTM 2XDENSE	Simulated only	Normalized	88.7%	0.889	0.892	0.887
3XCNN1D 1XLSTM 2XDENSE	Simulated only	Denoised	89.2%	0.894	0.897	0.892
3XCNN1D 2XLSTM 2XDENSE	Simulated only	Denoise Normalized	87.7%	0.879	0.882	0.877
3XCNN1D 1XLSTM 2XDENSE	IEMOCAP	Raw MFCC	82.3%	0.828	0.834	0.823
3XCNN1D 1XLSTM 2XDENSE	IEMOCAP	Normalized	83.6%	0.841	0.847	0.836
3XCNN1D 1XLSTM 2XDENSE	IEMOCAP	Denoised	81.6%	0.823	0.830	0.816
3XCNN1D 1XLSTM 2XDENSE	IEMOCAP	Denoise Normalized	81.6%	0.820	0.827	0.816
3XTDCNN1D 2XLSTM 2XDENSE	CREMAD	Raw MFCC	83.1%	0.833	0.839	0.831
3XCNN1D 1XLSTM 2XDENSE	CREMAD	Normalized	86.5%	0.868	0.875	0.865
3XCNN1D 1XLSTM 2XDENSE	CREMAD	Denoised	86.5%	0.867	0.872	0.865
3XCNN1D 1XLSTM 2XDENSE	CREMAD	Denoise Normalized	84.0%	0.843	0.848	0.840
3XTDCNN1D 2XLSTM 2XDENSE	RAVDESS	Raw MFCC	87.2%	0.873	0.875	0.872
3XTDCNN1D 2XLSTM 2XDENSE	RAVDESS	Normalized	82.4%	0.828	0.834	0.824
2XTDCNN1D 2XLSTM 2XDENSE	RAVDESS	Denoised	88.0%	0.882	0.886	0.880
3XCNN1D 1XLSTM 2XDENSE	RAVDESS	Denoise Normalized	84.7%	0.850	0.860	0.847
3XTDCNN1D 2XLSTM 2XDENSE	TESS	Raw MFCC	99.7%	0.997	0.997	0.997
3XTDCNN1D 2XLSTM 2XDENSE	TESS	Normalized	99.6%	0.996	0.996	0.996
DSCNN2D	TESS	Denoised	99.3%	0.993	0.994	0.993
2XTDCNN1D 2XLSTM 2XDENSE	TESS	Denoise Normalized	99.7%	0.997	0.997	0.997

**Table 3:** This table provides a brief comparison of other published algorithms using deep learning and similar databases. The asterisk (*) indicates the architectures investigated in this paper.

Architecture	TESS	RAVDESS	CREMA-D	IEMOCAP
3XTDCNN1D, 2XLSTM, 2XDENSE *	99.70%			
2XTDCNN1D, 2XLSTM, 2XDENSE *	99.70%			
3XTDCNN1D, 2XLSTM, 2XDENSE *	99.60%			
DSCNN2D *	99.30%			
1XANN, 1XPSO-FF [47]		88.70%		
2XTDCNN1D, 2XLSTM, 2XDENSE *		88.00%		
3XTDCNN1D, 2XLSTM, 2XDENSE *		87.20%		
3XCNN1D, 1XLSTM, 2XDENSE *			86.50%	
3XCNN1D, 1XLSTM, 2XDENSE *			86.50%	
3XDCNN, 2XLSTM [5]				86.16%
3XCNN1D, 1XLSTM, 2XDENSE *		84.70%		
3XCNN1D, 1XLSTM, 2XDENSE *			84.00%	
3XCNN1D, 1XLSTM, 2XDENSE *				83.60%
3XTDCNN1D, 2XLSTM, 2XDENSE *			83.10%	
2XCNN, BLSTM, 2XATTENTION [48]				82.80%
3XTDCNN1D, 2XLSTM, 2XDENSE *		82.40%		
3XCNN1D, 1XLSTM, 2XDENSE *				82.30%
3XCNN1D, 1XLSTM, 2XDENSE *				81.60%
3XCNN1D, 1XLSTM, 2XDENSE *				81.60%
6XDCNN [49]		75.83%		
6XDCNN [49]			65.77%	
2XVAE, 4XLSTM 2 [30]				64.93%
LSTM, ATTN/4, 3, 3, 4, 4, 4 [50]				63.50%
GAN, SVM [51]				60.29%
LSTM, MTL/3, 3, 2 [7]				56.90%
6XDCNN [49]	55.71%			
3XDCNN [52]				54.30%
2XLSTM, GAN [53]				53.76%
DCNN (VGG19), GAN/19 [54]				53.60%
CNN, VAE/5, 6, 4, 10, 5 [55]				48.54%

## Data Availability

Dataset available on request from the authors.
